# The Institutional Library

**Published:** 1916-12-02

**Authors:** 


### THE INSTITUTIONAL LIBRARY.

### HUMAN TEMPERAMENTS.

**Human Temperaments.** By CHARLES MERCIER, M.D., F.R.C.P. (London: The Scientific Press, Limited. 1916. Pp. 91. Price 1s. net.

DURING the publication in recent months of the unsigned articles on "Human Temperaments" which have appeared in the pages of THE HOSPITAL, there must have arisen speculations among our readers as to the identity of the writer of those incisive sketches of character. The secret is out now, at any rate; for in this republication of the articles in question, which numbers of readers will no doubt be glad of the opportunity of acquiring in collected form, Dr. Mercier's name appears upon the title-page. Once the disclosure is made, the first thing that will strike anyone acquainted with this distinguished author's contributions to medical literature is that no one else could possibly have been suspected. It is easy enough now to detect the Mercier touch in these mordant analyses of human greatness and littleness; but it may be doubted whether many of those who read them in their anonymous state realised exactly by whom they had been contributed to these columns. A detailed study of the essays would drive an acute observer to the inference that the author was probably a medical man, or failing that a solicitor—in either case of ripe experience and matured intellect. Once that has been taken for granted, it is almost legitimate to take the further step that the medical man is probably an alienist, or the solicitor has a large criminal practice, on the grounds that degenerate types—types of temperament leading, when exaggerated, to lunacy or crime—are described with greater fidelity and more searching insight than the higher types. Whether this is the case or not is, of course, a matter of opinion, upon which every reader is entitled to form his own views. In the opinion of the present reviewer, for what it is worth, the Suspicious Temperament and the Artistic Temperament are drawn truer to life than, say, the Practical Man and the Man of Action. This is not to say that the two latter are ill-drawn, but merely that as gems of analytical description they seem less flawless than the two former. For completeness with brevity, and for breadth of outlook with scrupulous accuracy in detail, the account of the Suspicious Temperament is a work of art in prose composition which has few superiors in our language. Possibly to any besides medical men and solicitors it may seem to be overdrawn; but any middle-aged member of either of these professions is certain to be struck by its amazing fidelity to fact in every particular.

In the chapter on the Religious Temperament Dr. Mercier employs all his powers of sarcasm and wit in picturing a type which is perhaps especially common in Great Britain as compared with Continental countries, though many of its exemplars succeed in deceiving the greater number of their contemporaries as to their real motives and nature. Whether there is another type of Religious Temperament besides that which Dr. Mercier has drawn with so slashing a technique, is a debatable question. Many people may be at first inclined to say that there is; and that the study here presented is incomplete. Dr. Mercier would perhaps reply that the commonest variety of the Religious Temperament is a weak and washy one, and that he has drawn the fully-developed but less common brand of it; and for this contention, that the Religious Temperament is one and not two, and that there is merely a wide fluctuation in the degree to which its characteristics are exhibited, there is undoubtedly much to be said. The distinction drawn between Cleverness and Capability is full of subtle understanding and deft portraiture; the likeness of the Faddist, in yet another chapter, may have been easier to draw, but the skill with which caricature has been avoided where exaggeration would have been so fatally facile is well worthy of notice.

That these lightning sketches of human temperament contain everything there is to be said on this almost inexhaustible topic the author, one may feel well assured, does not claim; but it is long since within so small a compass so much help was given to mankind in the pursuit of that "proper study," the knowledge of his fellow-man. Nor is it only the second half of the famous couplet which is quoted at the head of Dr. Mercier's preface that his pages help to promote. We defy anyone to read these essays on human temperament without qualms of self-examination and jars to his self-satisfaction. To him who desires to know himself, as well as to him who is more interested in his fellow-mortals, this collection of papers can alike be recommended for close study. Like all literature of the highest class, they will repay re-reading again and again; the opinion already expressed in this Journal that they reach a Thackerayan level is a compliment which we believe a good many critics will be found willing to endorse.

### WAR PROBLEMS

**Life Saving in War-time.** A Campaign Handbook. By MABEL PALMER, M.A. With an Introduction by the Right Hon. Arthur H. D. Acland. (London: C. Arthur Pearson, Ltd. Price 1s. net.)

THIS little book, with its warnings and suggestions, comes at an opportune time, and ought to prove most useful. The right note is struck in the Introduction, where we are warned to "look ahead." Whilst the war is destroying the very pick of our manhood, it is for those in authority to make a great effort in order to ensure that there shall be enough men and women in the future for war, to avert war, and to carry on successfully the industries of peace. This can only be done by rearing larger numbers of healthy children, and the object of this book is to show how and why the losses occur, how they can be largely prevented, and to stir up the country to prevent them.

The author shows us, firstly, the decrease of population by a lowered birth-rate—a loss of 467,837 infants between 1876 and 1914. The serious fact might also have been noted that this reduction is greatest amongst the better classes, those most capable of rearing their children and making of them healthy citizens. But, in addition to sterility, forced or otherwise, abortions and still-births have also to be reckoned with, the same conditions which cause them also frequently resulting in death during the early days of life. This is pointed out clearly, and as the death-rate is shown to be highest during the first three months of life, the value of ante-natal and early post-natal care is made evident.

Amongst the conditions causing these deaths—the incidence of which is very varied—are bad sanitation, polluted food, lack of care of the mother's own health, ignorance, poverty, carelessness, and vice. Although such conditions are to be found in rural as well as urban areas, yet it is town life which is most detrimental to the infant. The complicated nature of the whole question is well shown. The particularly fatal diseases of infancy which are dealt with can only be summarised here as those which show themselves at birth or shortly after, and which are largely due to ante-natal conditions; diarrhœa and other diseases due to improper feeding; measles and whooping-cough; bronchitis and pneumonia. Although the infant death-rate, which has been falling since the beginning of the twentieth century, shows, unfortunately, signs of again rising, yet Mrs. Palmer points out that if the matter were taken in hand more seriously by the Government, by local authorities, and by voluntary agencies, the annual holocaust of some 88,000 infants could be greatly reduced. Something is shown of what has already been accomplished and of what remains to be done. The Midwives Act was a great advance, but we are made to see that with the notified cases of puerperal fever still as high as 2,000 per annum there is little cause for congratulation, and large numbers remain unnotified. Careless midwifery is not confined to midwives, as Mrs. Palmer is brave enough to point out in her appeal for a larger supply of *well-trained* midwives and doctors. The four per 1,000 deaths of women from childbirth represent but a small amount of the ill-health both to mother and child which is left behind by this carelessness. The needs of the mother during pregnancy are dealt with. The suggestion of post-natal household help is an excellent one. If this were given also in special ante-natal cases the lives of many children and the lives and health of many mothers would undoubtedly be saved.

The Notification of Births Act gave public-health workers their first real chance of dealing with the death-rate rationally. The author enlarges upon the work of health visitors and infant-welfare centres, and recognises that in order to be successful the child should be dealt with from before birth until school age. A great incentive has been given to this work by Government grants.

One regrets that the most important question of all, the training of health visitors, should only have been dealt with seriously in a footnote on p. 95. Acknowledging that bad sanitation is one of the greatest enemies of infant life, a certificate of sanitary knowledge should surely be the foundation-stone of all training? This should be followed by some such scheme as that mentioned in the footnote. But thorough training has been wanted for many years, and not only because, as Mrs. Palmer states, "we are establishing a new kind of work for which special qualifications are really necessary." It is pathetic to note, from the character of their advertisements, that the only sort of woman some local authorities appear to require for their health visitor is a "fully trained nurse," one whose energies have been spent upon curing disease, and whose knowledge of preventive work is usually *nil.*

One cannot help criticising also the author's choice of illustrations in reference to rural and urban efforts. Warwickshire is not noticed, although that county has for some years been requiring from its health visitors at least a sanitary certificate, also that of the C.M.B., as well as previous experience in public-health work. Staffordshire is referred to at large, although it is proposing to employ as health visitors the nurses of the County Nursing Association, who, presumably, are still to act as midwives and who possess no sanitary training whatever.

Again, in dealing with voluntary efforts in London, all reference to the work of the City of Westminster Health Society is omitted. This Society was the first to organise health visiting in London, the first to use a Central Health Index, the first to link up infants with school children, and is now a training-school in practical work for health visitors. Nevertheless, Mrs. Palmer's book should prove a real incentive to further effort. Her enthusiasm alone must inspire others, and who could resist the appeal to be found in the statement that out of some 80,000 infants who must need help, only about 12,000 are at present dealt with?

**Our Hospital A B C.** Pictures by Joyce Dennys: Verse by Hampden Gordon and M. C. Tindall. (London: John Lane. 1916. Price 3s. 6d. net.)

There can, one imagines, be few military hospitals where a hospital alphabet (on the lines of "A was an archer," etc.) has not been constructed by some ingenious medical officer, sister, V.A.D., or orderly. Most of these productions are too ephemeral and too full of topical allusion and sly personalities to be of interest to anyone outside the particular institution in which they see the light. In this particular A B C, though there are obviously many topical jests contained in the verses or the drawings, they are not sufficiently obtrusive to spoil the humour for outsiders. In other words, they are capable of amusing the world at large as well as the colleagues of the collaborating authors. On the other hand, though the verses are good and the drawings better still, in neither is the very highest standard uniformly maintained; and this sort of fun requires to be extraordinarily good to be memorable. The medicine label on the first page is perhaps the happiest thought in the book, which is certain to obtain a welcome in auxiliary military hospitals.

**The Midwife's Pronouncing Dictionary.** Edited by Henry Robinson, M.A., M.D. (London: The Scientific Press, Limited. Price 1s. net.)

The second edition of this useful little manual has just been issued and brought completely up to date. The introduction of illustrations greatly enhances its value, and, though small, they are singularly clear and well chosen. The definition of every term a midwife is likely to come across is given (including "twilight sleep" under the heading of "Scopolamine"), and the Rules of the Central Midwives Board form a separate appendix. The whole book is but 3 inches by 4 inches, and not only pupil-midwives but those already in practice would do well to carry it about with them to study at odd moments.

